# The effect of temperature and methanol–water mixture on pressurized hot water extraction (PHWE) of anti-HIV analogoues from *Bidens pilosa*

**DOI:** 10.1186/s13065-016-0182-z

**Published:** 2016-06-08

**Authors:** Sefater Gbashi, Patrick Njobeh, Paul Steenkamp, Hlanganani Tutu, Ntakadzeni Madala

**Affiliations:** 1 Department of Biotechnology and Food Technology, Faculty of Science, University of Johannesburg, Doornfontein Campus, P.O. Box 17011, Johannesburg, Gauteng 2028 South Africa; 2 Department of Biochemistry, University of Johannesburg, P.O. Box 524, Auckland Park, Johannesburg, 2006 South Africa; 3 Council for Scientific and Industrial Research (CSIR), Biosciences, Natural Products and Agroprocessing Group, Pretoria, 0001 South Africa; 4 School of Chemistry, Molecular Sciences Institute, University of the Witwatersrand, WITS, Private Bag 3, Johannesburg, 2050 South Africa

**Keywords:** Pressurized hot water extraction, Co-solvent, *Bidens pilosa*, Dicaffeoylquinic acid, Chicoric acid, Response surface modeling

## Abstract

**Background:**

Pressurized hot water extraction (PHWE) technique has recently gain much attention for the extraction of biologically active compounds from plant tissues for analytical purposes, due to the limited use of organic solvents, its cost-effectiveness, ease-of-use and efficiency. An increase in temperature results in higher yields, however, issues with degradation of some metabolites (e.g. tartrate esters) when PHWE is conditioned at elevated temperatures has greatly limited its use. In this study, we considered possibilities of optimizing PHWE of some specific functional metabolites from *Bidens pilosa* using solvent compositions of 0, 20, 40 and 60 % methanol and a temperature profile of 50, 100 and 150 °C.

**Results:**

The extracts obtained were analyzed using UPLC-qTOF-MS/MS and the results showed that both temperature and solvent composition were critical for efficient recovery of target metabolites, i.e., dicaffeoylquinic acid (diCQA) and chicoric acid (CA), which are known to possess anti-HIV properties. It was also possible to extract different isomers (possibly *cis*-geometrical isomers) of these molecules. Significantly differential (p ≤ 0.05) recovery patterns corresponding to the extraction conditions were observed as recovery increased with increase in methanol composition as well as temperature. The major compounds recovered in descending order were 3,5-diCQA with relative peak intensity of 204.23 ± 3.16 extracted at 50 °C and 60 % methanol; chicoric acid (141.00 ± 3.55) at 50 °C and 60 % methanol; 4,5-diCQA (108.05 ± 4.76) at 150 °C and 0 % methanol; 3,4-diCQA (53.04 ± 13.49) at 150 °C and 0 % methanol; chicoric acid isomer (40.01 ± 1.14) at 150 °C and 20 % methanol; and *cis*-3,5-diCQA (12.07 ± 5.54) at 100 °C and 60 % methanol. Fitting the central composite design response surface model to our data generated models that fit the data well with R^2^ values ranging from 0.57 to 0.87. Accordingly, it was possible to observe on the response surface plots the effects of temperature and solvent composition on the recovery patterns of these metabolites as well as to establish the optimum extraction conditions. Furthermore, the pareto charts revealed that methanol composition had a stronger effect on extraction yield than temperature.

**Conclusion:**

Using methanol as a co-solvent resulted in significantly higher (p ≤ 0.05) even at temperatures as low as 50 °C, thus undermining the limitation of thermal degradation at higher temperatures during PHWE.

## Background

Plants constitute a vital part of the world’s primary health care [1]. *Bidens pilosa*, an underutilized plant species is a member of the Asteraceae family [2, 3] widely distributed around the world [4]. It is rich in phenolic compounds that are of great medical significance [5, 6]. More interestingly, *B. pilosa* has been shown to exhibit strong anti-HIV properties [7, 8]. As with other bioactive substances in plants, research is still ongoing to develop suitable techniques to extract these compounds from vegetal tissues. This continual quest for efficient and safe methods of extraction has propelled the evolution and adoption of pressurized hot water extraction (PHWE). Conventional organic solvent extraction techniques elicit issues of safety, they are laborious and also time-consuming [9, 10]. Often referred to as subcritical water extraction [11], PHWE is an efficient and greener method for the extraction of bioactive compounds from plant materials [10, 11]. It is particularly advantageous because water is readily available, non-toxic, non-flammable, and environmentally friendly [12]. Moreover, PHWE is a less sophisticated and an easy-to-use technology, requiring less time and expertise compared to conventional methods of extraction [13].

However, a major setback to this ingenious system has been the thermal degradation phenomenon observed at elevated temperatures for certain compounds [14–17], hence the need for optimization [18]. Amidst possible optimization approaches [19, 20], the principle of co-solvency seems particularly promising in terms of enhanced extraction efficiency [21–24]. Accordingly, methanol has been recommended for pressurized liquid extraction [25]. It is 100 % miscible with water and has a high solvation power for marker compounds compared to other solvents [26, 27]. A study comparing the effectiveness of methanol and ethanol as cosolvents during supercritical fluid extraction have also reported the superior performance of methanol over ethanol [28]. This was also corroborated by Pinho and Macedo who observed that water–methanol mixture had a higher solvation power than its corresponding ethanol counterpart [29]. Furthermore, methanol is cheaper and readily available, thus could offer a good option as a cosolvent during PHWE. In this study, we investigated the effect of different compositions of methanol–water mixture and temperature conditions on PHWE of different isomers of diCQA and chicoric acid (CA) (anti-HIV analogues) from stem and leaves of an underutilized plant, *B. pilosa*.

## Experimental section

### Plant materials and metabolite extraction

**Fig. 1:**
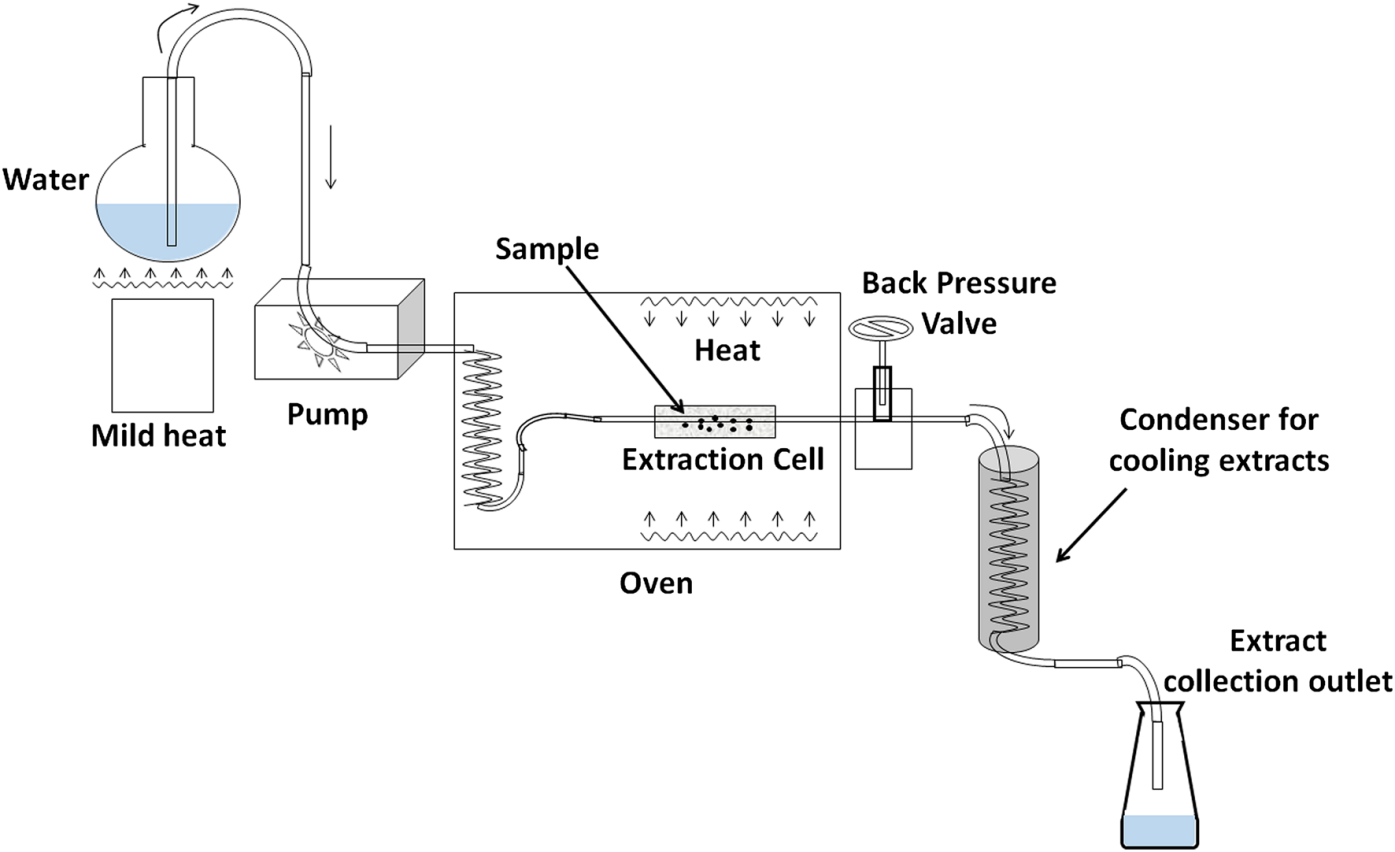
Diagrammatic representation of our PHWE unit

*Bidens pilosa* plants were collected from the Venda region of Limpopo province (South Africa). Sample preparation and extraction followed procedures described by Khoza et al. [14]. The plant materials were air-dried (10 % moisture content) at ambient conditions in a dark and well-ventilated room for 7 days after which, they were crushed to powder (≤0.5 mm) using a mortar and pestle. Extraction of phytochemicals was achieved by a makeshift laboratory scale PHWE unit (Fig. 1). The system consisted of a HPLC pump (Waters 6000 fluid controller, Waters Corporation, Manchester, UK), stainless steel extraction cell (70 × 30 mm and approximately 20 mL) fitted with a metal frit i.e. filter (3/8 in. diameter, 1/32 in. thickness and 2.0 µm pore size), refurbished GC 600 Vega Series 2 oven (Carlo Erba Instruments, Italy) with an automatic temperature controllable unit, stainless tubing (1.58 mm in outer dimension (OD) and 0.18 mm inner dimension (ID), back-pressure valve (Swagelok, Johannesburg, South Africa), and a collection flask.

For the extraction, 4 g of ground leaves powder was mixed with 2 g of diatomaceous earth (Sigma, Munich, Germany), a dispersing agent and placed inside the extraction cell maintained at different oven temperatures of 50, 100 and 150 ± 1 °C. Extraction was performed in dynamic mode using different ratios of methanol–water mixture i.e. 0, 20, 40 and 60 % composition of aqueous methanol (Romil Ltd, Waterbeach Cambridge). The solvent was delivered at a constant flow rate of 5 mL/min and a pressure of 1000 ± 200 psi was maintained using the back-pressure valve. Extracts were collected in a falcon tube up to the 50 mL mark through an outlet coil immersed in a cooling water bath. Each extraction operation lasted for 10 min. The extracts were filtered using a 0.22 µm nylon syringe filter into a 2 mL HPLC capped vial and preserved at −20 °C prior to analysis.

### Chromatographic separation and mass spectrometry (UPLC-qTOF-MS)

The chromatographic separation was performed on a UPLC hyphenated to a Synapt G1 -qTOF-MS instrument (Waters Corporation, Manchester, UK) equipped with a Waters Acquity HSS T3 C_18_ column (150 × 2.1 mm diameter and particle size 1.8 µm). The column oven temperature was maintained at 60 °C. The mobile phases were (A) 0.1 % formic acid in deionized water, and (B) mass spectrometry (MS)-grade acetonitrile with 0.1 % formic acid. The linear gradient program began with 2 % A to 60 % B for 24 min, ramped to 95 % B at 25 min and kept constant for 2 min, then re-equilibrated at 5 % B for 3 min. The total cycle runtime was 30 min with a flow rate of 0.4 mL/min.

Mass spectrometry was performed using a Waters qTOF-MS instrument (Waters Corporation, Manchester, UK) fitted with an electrospray ionization source (ESI) operating in both positive and negative ion electrospray modes. The *m/z* range was 100–1000, scan time 0.2 s, interscan delay 0.02 s, with leucine encephalin (556.3 µg/mL) as a lock mass, standard flowrate 0.1 mL/min, and a mass accuracy window of 0.5 Da was used for MS data acquisition. Moreover, the instrument was operated on the following settings: collision energy of 3 eV, capillary voltage of 2.5 kV, sample cone voltage of 30 V, detector voltage of 1650 V (1600 V in negative mode), source temperature at 120 °C, cone gas flow at 50 (L/h), and desolvation gas flow at 550 (L/h). To achieve metabolite fragmentation patterns necessary for annotation or identification, the collision energy during MS acquisition was experimentally changed in the trap ion optics by acquiring data at 3, 10, 20 and 30 eV.

### Data analyses

Data acquired was analyzed and visualized using Markerlynx XS software (Waters Corporation, Manchester, UK). For maximum data output, the analysis was carried out using optimized parameters [14]. Here, only negative data were analyzed using similar optimized parameters, for reasons of better predictability without need for use of authentic standards [14, 30]. Representative single ion monitoring (SIM) chromatograms for target molecules were generated using their *m/z* values. Moreover, various MS spectra for these molecules were obtained from the chromatograms, their fragmentation patterns observed, and molecular formulae calculated on the basis of a 5 ppm mass accuracy range. This information was used to confirm the identities of these bio-markers following a search of the Dictionary of Natural Products online database [31] in an approach previously reported [14].

Extraction yields for molecules identified represented the relative peak intensity figures of molecular peaks corresponding to the identified molecules. Relative peak intensity is a dimensionless quantity, and corresponded to the area-under-the-peak values obtained from the peak list. This data file (peak list) is the final output obtained after processing of the MS data using MarkerLynx software [32, 33].

### Statistical analysis

A one-way analysis of variance (ANOVA) was performed on data obtained from Markerlynx XS software and the mass distribution patterns of the means graphically described by the Box-and-Whisker plots. Duncan’s multiple comparison test was performed using ANOVA to determine the differences between individual extraction conditions using IBM SPSS software version 22 (SPSS/IBM, Chicago, Illinois) [34–36]. Mean values of extraction conditions were deemed to be different if the level of probability was ≤0.05.

The central composite design response surface model (CCD RSM) was fitted to experimental data in order to obtain the relationship between factors and optimize the response of Z (metabolite yield) in relation to X (solvent composition) and Y (extraction temperature) using Statistica rel 7 (StatSoft, USA) [37]. By using CCD, a total of 12 experimental runs (including 3 repetitions) were designed, 3 factor levels for temperature (50, 100, 150 °C) conditions and 4 factor levels for solvent composition (0, 20, 40 and 60 % methanol). In order to optimize the response, it was essential for quadratic terms to be included in the polynomial function (i.e. a second-order polynomial model) represented by the form of Eq. 1:

1 $${\rm z} ( {\rm x},{\rm y} )  = {\rm c}_{00} + {\rm c}_{10} {\rm x} + {\rm c}_{ 20} {\rm x}^{ 2} + {\rm c}_{0 1} {\rm y} + {\rm c}_{0 2} {\rm y}^{ 2} + {\rm c}_{ 1 1} {\rm xy}$$

In this case, Z was the dependent variable/predicted response factor, and X and Y the independent variables, c_00_ is a constant, c_10_ and c_01_ are the linear coefficients of X and Y, respectively, c_20_ and c_02_ are the quadratic coefficients of X and Y, respectively, and c_11_ is the interaction coefficient. Equation 1 was fitted to experimental data by using a statistical multiple regression approach called method of least square (MLS), which generates the lowest possible residual [38]. Model parameters and model significance were determined at p < 0.05. The fitness of the model was determined by evaluating the coefficient of regression (R^2^) obtained from the analysis of variance (ANOVA). The model fit generates the response surface that defines the behaviour of the response variable, which can be conveniently visualized on the surface plot and contour plot. By means of these plots, the optimized ranges for each factor (i.e. temperature and methanol composition) that leads to the highest response (metabolite yield) can be extracted [38, 39].

## Results and discussion

**Fig. 2:**
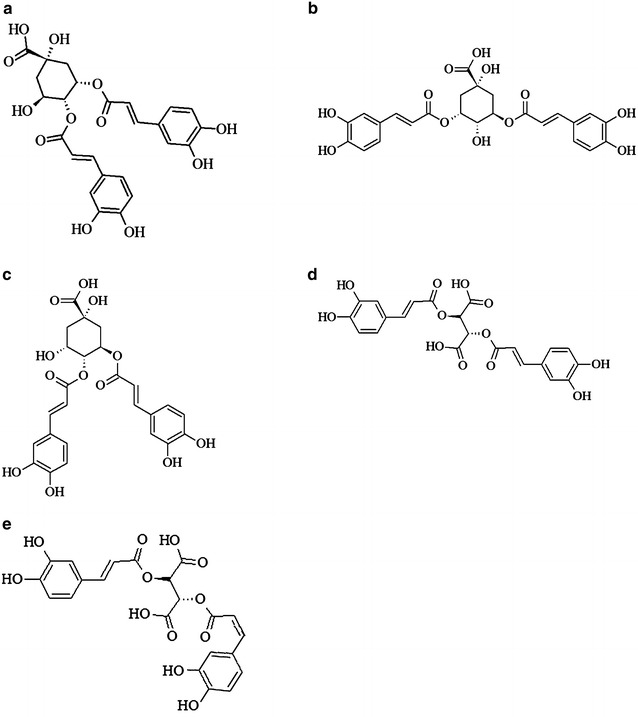
Molecular structures of 3,4 diCQA (**a**), 3,5 diCQA (**b**), 4,5 diCQA (**c**), CA (**d**) and CA isomer (**e**)

**Fig. 3:**
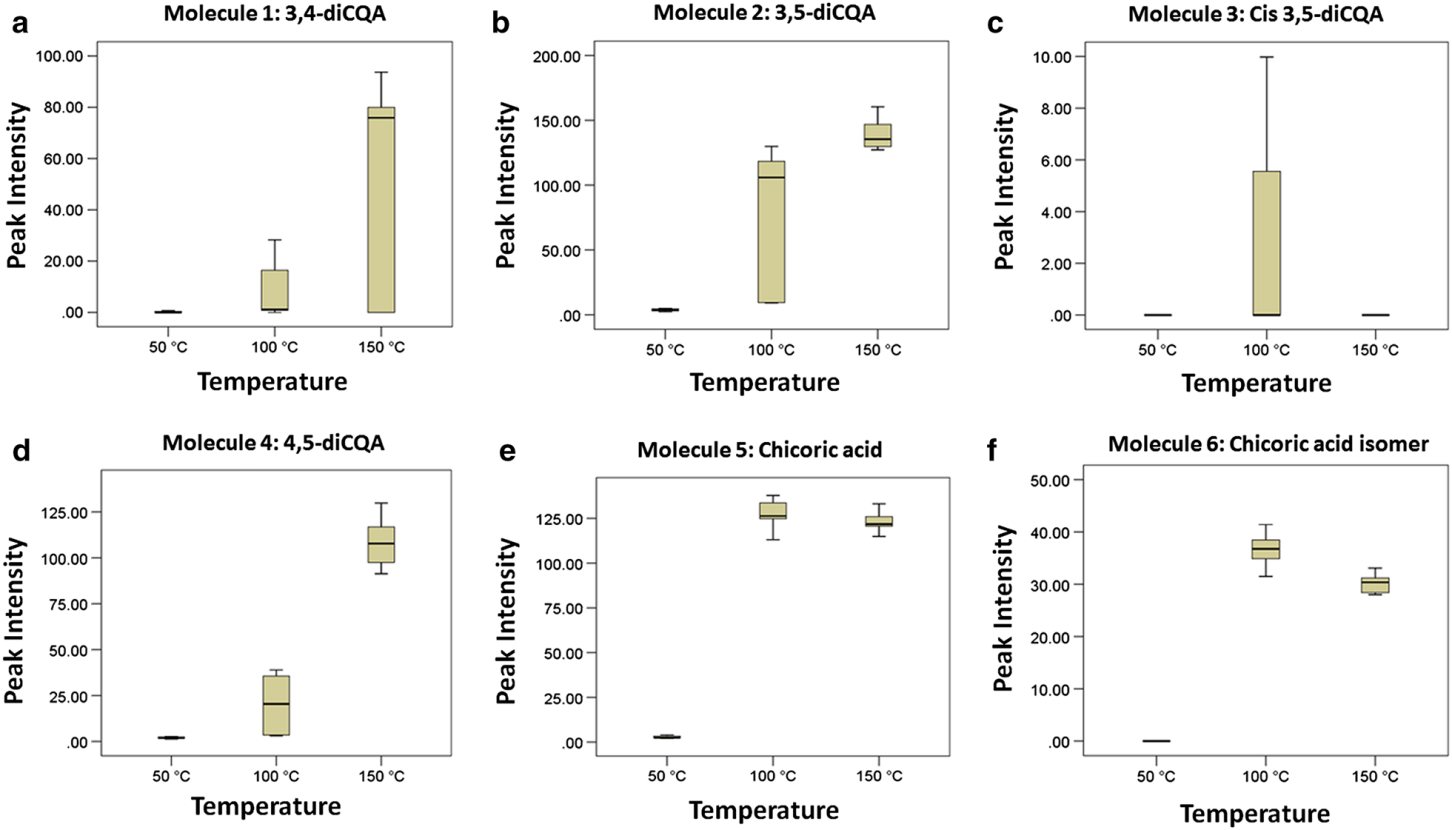
*Box*-and-*whiskers* plots showing the effect of temperature on the extractability of isomers of diCQA and CA using water-only PHWE: 3,4-diCQA (**a**), 3,5-diCQA (**b**), *Cis*-3,5-diCQA (**c**), 4,5-diCQA (**d**), CA (**e**) and CA isomer (**f**)

**Fig. 4:**
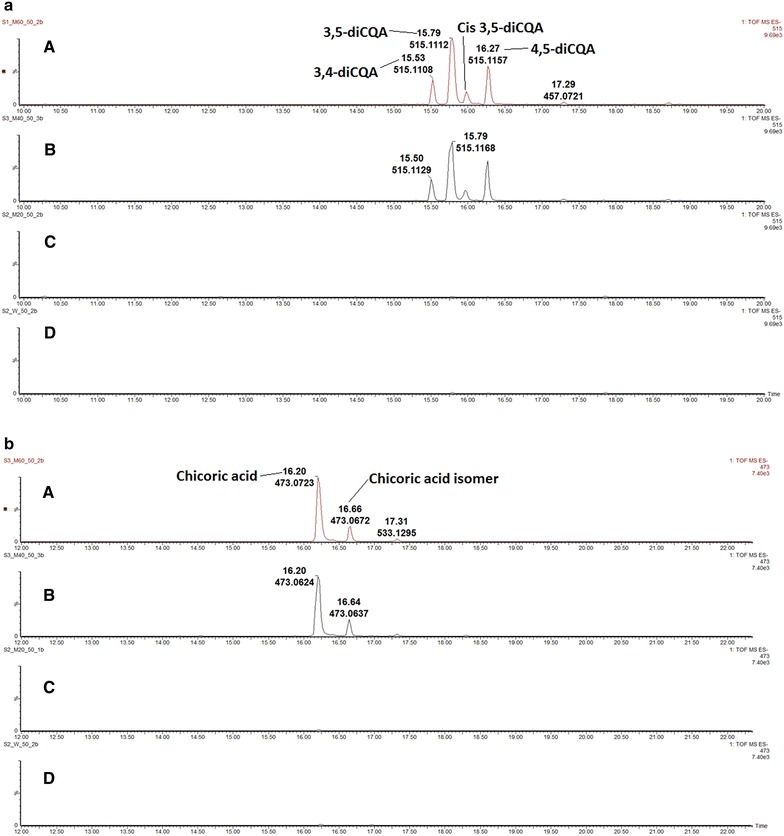
**a** Representative UPLC-MS single ion monitoring (SIM) chromatograms for isomers of diCQA following PHWE of *B. pilosa* at 50 °C using 60 % MeOH (*A*), 40 % MeOH (*B*), 20 % MeOH (*C*) and 0 % MeOH (water) (*D*). **b** Representative UPLC-MS single ion monitoring (SIM) chromatograms for chicoric acid and chicoric acid isomer following PHWE of *B. pilosa* at 50 °C using 60 % MeOH (*A*), 40 % MeOH (*B*), 20 % MeOH (*C*) and 0 % MeOH (water) (*D*)

**Fig. 5:**
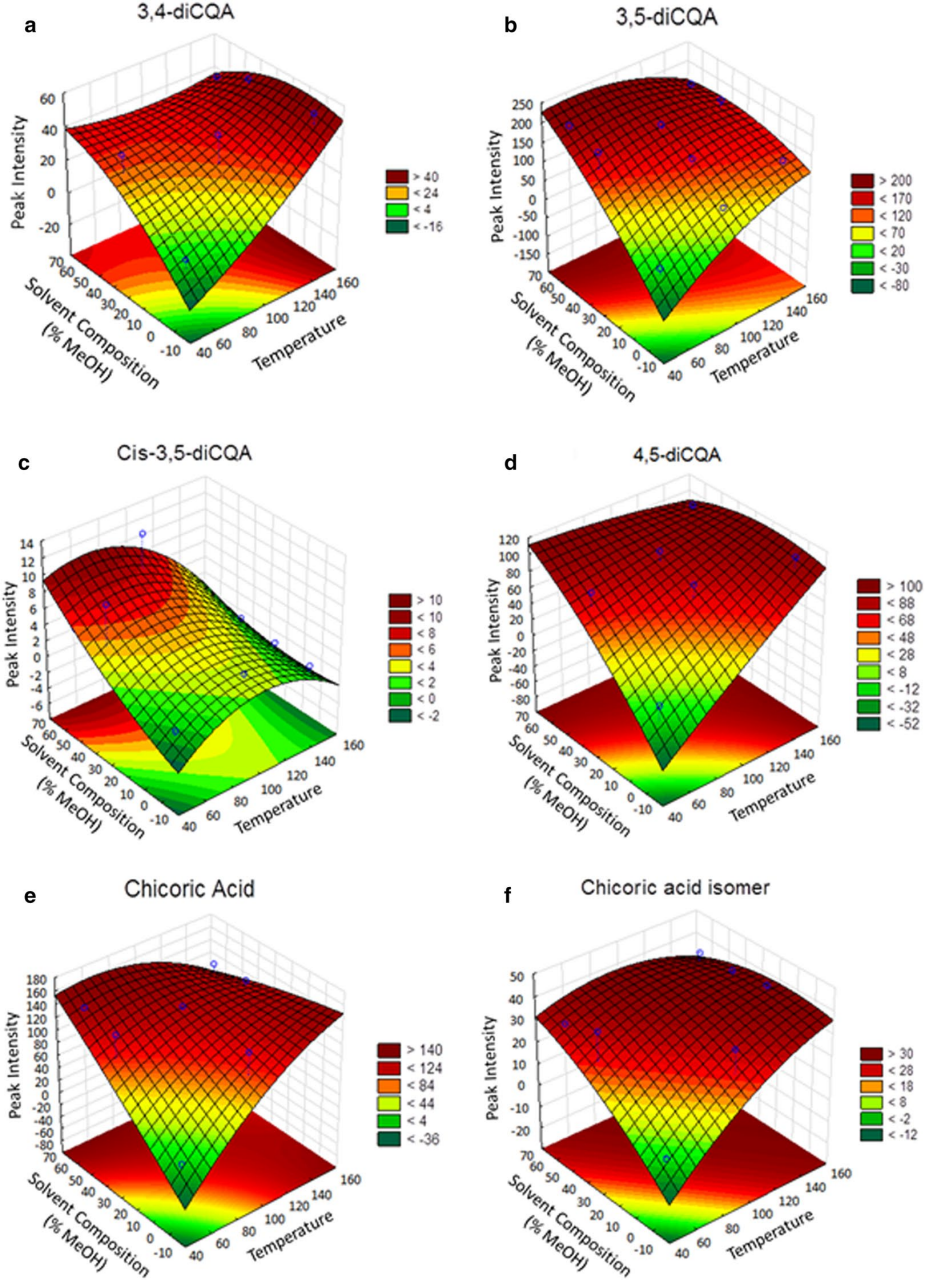
Surface plots showing the effect of temperature and solvent composition on the extraction of diCQA and CA analogues: 3,4-diCQA (**a**), 3,5-diCQA (**b**), *Cis*-3,5-diCQA (**c**), 4,5-diCQA (**d**), CA (**e**) and CA isomer (**f**)

**Fig. 6:**
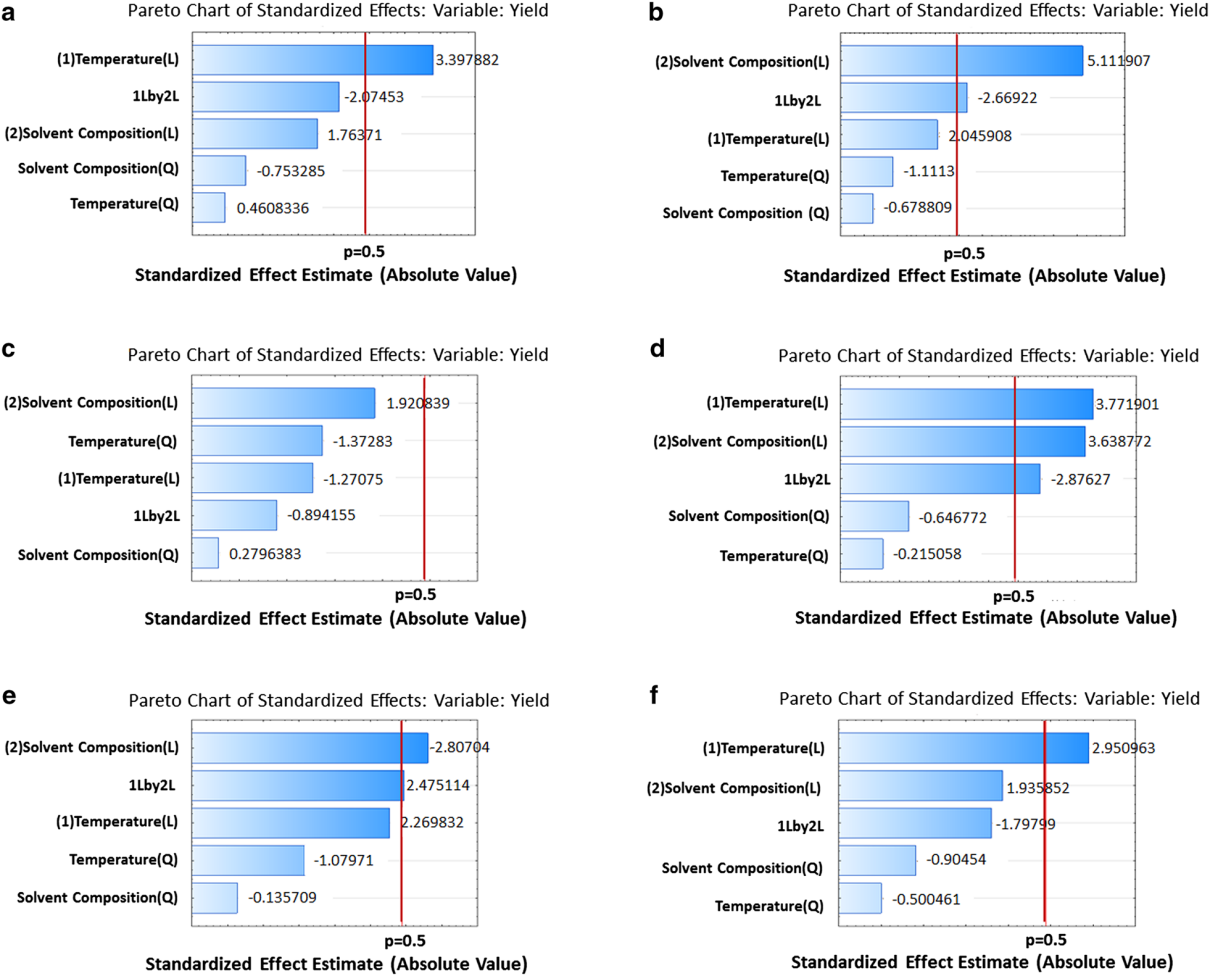
Pareto chart of standardized effects of temperature and solvent composition on the extraction of diCQA and CA analogues: 3,4-diCQA (**a**), 3,5-diCQA (**b**), *Cis*-3,5-diCQA (**c**), 4,5-diCQA (**d**), CA (**e**) and CA isomer (**f**)

*Bidens pilosa* is rich in bioactive compounds that are of great medicinal significance [5, 6]. In this study, we demonstrated the extraction of functional metabolites (specifically anti-HIV analogues) from this plant using a modified PHWE approach. The PHW was modified using different compositions of methanol–water mixture (0, 20, 40 and 60 % methanol), and the effect of solvent composition and extraction temperature (50, 100 and 150 °C) on the recovery of target metabolites was investigated. Various isomers of diCQA and CA were successfully extracted. The presence of these metabolites in *B. pilosa* and closely related species have been reported in the literature [5, 40]. Using a sensitive and robust tandem MS approach with settings presented elsewhere [41], it was possible to conveniently fingerprint these molecules. Table 1 and Fig. 2 show the different fragmentation patterns and structural configurations of these metabolites, meanwhile their patterns of recovery are provided in Table 2 and Figs. 3, 4, 5, 6.

**Table 1:** Identified metabolites extracted from *B. pilosa* by PHWE

Mol. #	Mol. name	Rt	*m/z*	MS fragments
1	3,4-diCQA	15.53	515	353, 191, 173, 179, 135
2	3,5-diCQA	15.79	515	191, 179, 135
3	*Cis*-3,5-diCQA	15.98	515	191, 179, 135
4	4,5-diCQA	16.27	515	353, 191, 173, 179, 135
5	CA	16.20	473	311, 293, 179, 149, 135
6	CA Isomer	16.64	473	311, 293, 179, 149, 135

*Mol* molecule; *Rt* retention time; *m/z* mass to charge ratio

**Table 2:** Yield (mean relative peak intensity) of identified anti HIV analogues extracted from *B. pilosa* using modified PHWE

Parameters	3,4-diCQA	3,5-diCQA	*Cis*-3,5-diCQA	4,5-diCQA	CA	CA isomer
T_50_C_0_	0.21 ± 0.09^a^	3.70 ± 0.33^a^	0.00 ± 0.00^a^	2.00 ± 0.15^a^	2.82 ± 0.23^a^	0.00 ± 0.00^a^
T_50_C_20_	1.91 ± 0.93^a^	18.30 ± 8.02^a^	0.36 ± 0.36^a^	3.21 ± 0.88^a^	19.85 ± 8.57^a^	2.27 ± 1.14^a^
T_50_C_40_	37.12 ± 9.42^b,c^	188.24 ± 2.48^e^	9.64 ± 4.86^b,c^	85.13 ± 2.21^c^	131.47 ± 3.65 ^c,d^	36.27 ± 1.97^b,c,d^
T_50_C_60_	32.54 ± 10.41^b,c^	*204.23* ± *3.16* ^e^	4.01 ± 3.67^a,b,c^	91.71 ± 2.10 ^c,d^	*141.00* ± *3.55* ^d^	30.13 ± 0.55^b,c^
T_100_C_0_	10.18 ± 4.04^ab^	80.05 ± 17.96^b^	2.95 ± 1.26^a,b^	20.59 ± 5.00^b^	127.02 ± 2.76^b,c,d^	34.36 ± 2.62^b,c,d^
T_100_C_20_	44.93 ± 8.51^c^	150.46 ± 16.03 ^c,d^	0.10 ± 0.10^a^	84.22 ± 1.31^c^	105.23 ± 18.68^b^	28.77 ± 5.67^b^
T_100_C_40_	26.98 ± 10.69^a,b,c^	186.70 ± 4.43^e^	4.56 ± 3.97^a,b,c^	99.97 ± 3.11^d,e^	130.53 ± 3.00 ^c,d^	33.55 ± 2.82^b,c,d^
T_100_C_60_	32.90 ± 10.42^b,c^	185.83 ± 2.99^e^	*12.07* ± *5.54* ^c^	96.93 ± 2.81^d^	111.86 ± 13.39^b,c^	30.87 ± 4.78^b,c^
T_150_C_0_	*53.04* ± *13.49* ^c^	124.75 ± 15.97^c^	0.00 ± 0.00^a^	*108.05* ± *4.76* ^d,e^	121.66 ± 2.86^b,c,d^	30.19 ± 0.60^b,c^
T_150_C_20_	38.77 ± 12.29^b,c^	127.69 ± 19.96^d,c^	0.16 ± 0.16^a^	93.67 ± 3.40^d^	126.17 ± 2.23^b,c,d^	*40.01* ± *1.14* ^d^
T_150_C_40_	51.35 ± 9.81^c^	181.25 ± 4.98^d,e^	0.76 ± 0.26^a^	98.48 ± 2.55^d^	128.33 ± 2.51^b,c,d^	38.03 ± 0.59 ^c,d^
T_150_C_60_	41.54 ± 10.46^c^	175.11 ± 2.64^de^	0.07 ± 0.0^7a^	99.66 ± 2.21^d,e^	123.48 ± 2.28^b,c,d^	37.37 ± 0.55 ^c,d^
Level of significance	***	***	**	***	***	***

Values represent means of triplicate extraction yield ± SEM (standard error of the mean). Values within the same column followed by different superscripts are significantly different (p < 0.05). Level of significance *** p < 0.001, and ** p < 0.01. Values in italics (within a *column*) represent the highest extraction yields for the molecule

T_50_C_0_—extraction at 50 °C and 0 % methanol; T_50_C_20_—extraction at 50 °C and 20 % methanol; T_50_C_40_—extraction at 50 °C and 40 % methanol; T_50_C_60_—extraction at 50 °C and 60 % methanol; T_100_C_0_—extraction at 100 °C and 0 % methanol; T_100_C_20_—extraction at 100 °C and 20 % methanol; T_100_C_40_—extraction at 50 °C and 40 % methanol; T_100_C_60_—extraction at 50 °C and 60 % methanol; T_150_C_0_—extraction at 50 °C and 0 % methanol; T_150_C_20_—extraction at 50° °C and 20 % methanol; T_150_C_40_—extraction at 50 °C and 40 % methanol; T_150_C_60_—extraction at 50 °C and 60 % methanol

In view of that, Molecules 1-4 were identified as isomers of dicaffeoylquinic acid (diCQA) i.e. 3,4-diCQA, 3,5-diCQA, *cis*-3,5-diCQA, and 4,5-diCQA, respectively, by their parent ion peak (in negative ionization mode) at *m/z* 515 with fragment ions at *m/z* 353, 191, 179, 173 and 135 [41, 42]. These isomers were further distinguished by their order of elution and patterns of fragmentation as reported by these authors [43–45]. Molecules 5 and 6 were identified as chicoric acid (CA) and CA isomer, with a parent ion peak at *m/z* of 473, and MS2 base peak ion at *m/z* of 311 (for di-caffeoyltartaric acid) due to the loss of a hexose (162 Da), and other fragments at *m/z* 179 (caffeic acid), and 149 (tartaric acid) [46, 47]. diCQA and CA have been widely reported to exhibit anti-HIV properties via the inhibition of HIV-1 integrase. Interestingly, these compounds have lethal doses that are multiple-times (at least 100-fold) above their antiviral concentrations [48].

Figure 3 shows the box-and-whiskers plots of the effect of temperature on the extractability of target metabolites (molecules 1–6) using non-modified (i.e. water only) PHWE. From these plots, it was clearly evident that PHWE was applicable for the extraction of diCQA and CA and their analogues, and that temperature played a key role in the recovery patterns of these molecules. It can be seen that extraction yield increased substantially with increase in temperature. 3,4-diCQA increased from 0.21 (50 °C) to 53.04 (150 °C), a 252-fold increase in recovery corresponding to a 100 °C increase in temperature. Similarly, 3,5-diCQA and 4,5-diCQA increased by magnitudes of 33.72 and 54.03, respectively, following an increase in temperature from 50 to 150 °C.

The observed enhancement of recovery efficiency with increase in temperature can be attributed to the alteration of the properties of water at elevated temperatures. As the temperature of pressurized water increases its dielectric constant, viscosity and surface tension decreases, while its diffusivity increases [10, 49]. Moreover, the thermal energy supplied can overcome cohesive (solute–solute) and adhesive (solute–matrix) interaction by decreasing the activation energy required for the desorption process [49]. Additionally, the high pressures involved in PHWE can facilitate extraction by forcing the fluid into areas of the sample matrix that would not normally be contacted by fluid under atmospheric pressure [50].

Although temperature was found to be critical during PHWE of *B. pilosa*, the positive effect of temperature on the extractability of *cis*-3,5-diCQA, CA and CA isomer occurred only between temperatures of 50 and 100 °C. At a temperature of 150 °C there was a decrease in extraction yield for these molecules which can be attributed to thermal degradation. It is common knowledge that during PHWE, higher temperatures degrade some classes of plant metabolites [14, 15]. This degradation phenomenon is a major limitation of PHWE. Moreover, it was apparent that target metabolites were only fairly soluble in low temperature water (50 °C). Hence, it became necessary to optimize the PHWE method for a more efficient and safe recovery of these metabolites. In this regard, methanol was added as a cosolvent during PHWE of target metabolites from *B. pilosa* and the results presented (Fig. 4a, b; Table 2). Figure 4a and b shows the extractability of target metabolites using (a) 0 % methanol, (b) 20 % methanol, (c) 40 % methanol and (d) 60 % methanol, at 50 °C on single ion monitoring (SIM) chromatograms. From the visual evaluation of these chromatograms, it is clearly evident that incorporation of methanol significantly enhanced the recovery of diCQA, CA and their analogues during PHWE of *B. pilosa*.

The enhancement in extraction efficiency was both qualitative (number of components) and quantitative, and also in proportions to the percentage of methanol composition as was apparent from the base peak ion (BPI) chromatograms (not shown) and from the intensity of colour of the extracts (not shown). Table 2 shows the extraction yield obtained at various extraction conditions of temperature and solvent composition. These results indicate that extraction conditions (temperature and solvent composition) resulted in significantly different (p ≤ 0.05) recovery patterns for each metabolite (Table 2). It was also possible to show the main compounds recovered and in descending order of yield, they include 3,5-diCQA with a yield of 204.23 ± 3.16 extracted at 50 °C and 60 % methanol; chicoric acid (141.00 ± 3.55) at 50 °C and 60 % methanol; 4,5-diCQA (108.05 ± 4.76) at 150 °C and 0 % methanol; 3,4-diCQA (53.04 ± 13.49) at 150 °C and 0 % methanol; chicoric acid isomer (40.01 ± 1.14) obtained at 150 °C and 20 % methanol; and *cis*-3,5-diCQA (12.07 ± 5.54) obtained at 100 °C and 60 % methanol (Table 2).

Essentially, the adoption of methanol as a co-solvent during PHWE made it possible to achieve significantly (p ≤ 0.05) higher extraction yields even at a low temperature of 50 °C, which was heretofore, unachievable even when temperatures were raised to 150 °C using water only. For example, at a constant temperature of 50 °C, the extraction yield of 3,5-diCQA increased by a factor of 55.2 as methanol composition rose from 0 % methanol (water only) to 60 % methanol. Likewise, CA increased by a factor of 50 from 0 % methanol to 60 % methanol, under similar temperature conditions. This is in agreement with the earlier report of Ong et al. [51] who observed that at constant temperature, a better extraction efficiency could be achieved by increasing the amount of ethanol added in the water (0–30 %), during the pressurized liquid extraction of tanshinone IIA in *Salvia miltiorrhiza*.

Particularly, the efficient recovery of CA at low temperatures is very interesting and desirable because, this compound is known to be highly unstable and degrade rapidly during the extraction process [9, 52, 53]. This metabolite has been proposed as an indicator compound for quality control due to its instability and rapid degradation when compared to other secondary metabolites within plant materials [9, 18]. Enhancement due to the incorporation of methanol as a cosolvent during PHWE can be associated with interactions based on polarity. As organic compounds diCQA and CA are highly soluble in organic solvents such as methanol, and were favoured by higher percentages of methanol. The presence of methanol in water greatly reduced the polarity of water without a need for increasing the temperature. Moreover, as compared to pure water, water–methanol mix is a less dense solvent mixture which has lower surface tension, lower hydrogen bonding strength between water molecules and higher diffusivity [10]. As such during extraction, there was a higher permeability into the cellular structures of the matrix, which resulted in better extractability.

Also, it was observed that the gradient increase in extraction yield due to incorporation of methanol as a cosolvent during PHWE was more steep (rapid) at low temperatures compared to higher temperatures. For example, when comparing the rate of increase from 0 % methanol to 60 % methanol, 4,5-diCQA increased by a factor of 45.86 at 50 °C, 4.71 at 100 °C, and 0.92 at 150 °C (Table 2). Moreover, at higher temperatures (150 °C), there was a slight decrease in recovery efficiency as methanol composition increased. We saw that for all extractions obtained at 150 °C, the highest yields were obtained at 40 % methanol rather than the expected 60 % methanol. To give an instance, the recovery of CA rather decreased by 3.78 % when methanol composition was increased from 40 to 60 % during extraction at 150 °C. The reason for this phenomenon is unclear and requires further investigation.

In order to better interpret and describe the patterns in our data set, we adopted the central composite design response surface methodology (CCD RSM) statistical approach. Response surface methodology is an ideal statistical approach to employ when a response or a group of responses of interest are influenced by more than one variable [54]. In our case, extraction yield was influenced by temperature and solvent composition. Accordingly, the CCD RSM was fitted to the experimental data with R^2^ values ranging from 0.57 to 0.87, implying that the fit explains 57–87 % variability in the response variable. Coefficient of determination (R^2^) values above 0.70 indicates a model that fits the data well. Three dimensional surface plots were generated from the model fit in order to conveniently visualize the interrelationship of the levels of factors and the recovery patterns of target metabolites (Fig. 5). From these plots again, it was visibly evident that temperature and more profoundly methanol composition were critical for the efficient extraction of different isomers of diCA and CA. The colour bands on the smooth surface corresponds to the response of the dependent variable relative to the levels of the independent variables such that, regions with dark green colour represent low extraction yields, while those regions with dark red colour represent high extraction yield. Hence, it was possible to determine regions with the most efficient performance of the system through visual inspection of the surfaces. Equations 2–7 represent the response surface equations for molecules 1–6 in that order.

2 $$\begin{aligned}{\rm z}& = - 19.92745 + 0.24828 {\rm x} + 0.00133 {\rm x}^{2} + 1.42157 {\rm y} \\ & \quad-0.00639 {\rm y}^{ 2} - 0.00771 {\rm xy} + 0 \end{aligned}$$

3 $$\begin{aligned}{\rm z} &= - 1 4 7. 3 3 8 7 3 + 3. 1 6 4 4 7 {\rm x} - 0.00 9 1 4 {\rm x}^{ 2} + 6.0 3 7 8 9 {\rm y} \\ &\quad-0.0 1 6 4 5 {\rm y}^{ 2} - 0.0 2 8 3 5 {\rm xy} +0\end{aligned}$$

4 $$\begin{aligned}{z} &= - 9. 4 8 60 6 + 0. 2 4 1 9 1 {\rm x} - 0.00 1 2 2 {\rm x}^{ 2} + 0. 1 4 8 4 6 {\rm y} + 0.000 7 3 {\rm y}^{ 2} \\ &\quad- 0.00 10 3 {\rm xy} +0 \end{aligned}$$

5 $$\begin{aligned}{\rm z} &=- 7 8.0 8 3 6 6 + 1. 3 1 6 80 {\rm x} - 0.00 10 8 {\rm x}^{2} +  3. 3 8 80 5 {\rm y} \\ &\quad-0.00 9 5 3 {\rm y}^{ 2} - 0.0 1 8 5 7 {\rm xy} + 0 \end{aligned}$$

6 $$\begin{aligned}{\rm z } &= - 1 1 4. 3 2 8 9 1 +  2. 8 3 4 2 1 {\rm x } - 0.00 7 7 3 {\rm x}^{ 2} + 3. 6 20 5 5 {\rm y }\\ &\quad - 0.00 2 8 6 {\rm y}^{ 2} - 0.0 2 5 9 3 {\rm xy } + 0\end{aligned}$$

7 $$\begin{aligned}{\rm z } &= - 3 1. 7 3 4 2 6  + 0. 7 5 80 4 {\rm x } - 0.00 20 4 {\rm x}^{ 2} + 0. 9 5 4 2 2 {\rm y } \\ &\quad- 0.00 3 3 3 {\rm y}^{ 2} - 0.00 5 2 4 {\rm xy } + 0 \end{aligned}$$

where x = methanol composition; y = temperature; z = extraction yield

Furthermore, the model fit afforded insights on the patterns of distinct variable effects and pairwise (mutual) variables interactive effects on the response variable (Fig. 6). Figures 5 and 6 show the pareto charts of standardized factor effects from which the magnitude and importance of each effect (p ≤ 0.05) can be envisaged. The reference line indicated on the chart (α = 0.05) distinguishes between significant and insignificant effects, such that any effect that extends beyond this reference line is significant [55]. As such, the linear effect of temperature had the highest impact on extraction yield for 3,4-diCQA, followed by the interactive effect of temperature and solvent composition, the linear effect of solvent composition, the quadratic effect of solvent composition, and the quadratic effect of temperature. Linear effect of a variable means that the variable correlates directly proportional to the response variable, whereas the quadratic effect of a variable implies that the response variable is correlated with the square of that variable.

A strong quadratic effect of a variable (p < 0.05) implies that the optimal levels of the response falls within the range of the experimental values for that variable, and vice versa. From the fitted models, none of the quadratic effects was significant (p ≤ 0.05), which implies that all optimal extraction conditions fall outside the experimental domain. 3,5-diCQA had the weakest quadratic effect on solvent composition, which implies that this molecule has the highest solubility in methanol (an indication of high polarity). Moreover, it can also be seen that temperature and solvent composition had a significant (p ≤ 0.05) synergistic effect on the recovery patterns of 3,5-diCQA, 4,5-diCQA, and CA (Fig. 6). In general, solvent composition had a higher impact on the recovery efficiency of target metabolites than temperature.

## Conclusion

Using a modified PHWE approach, we demonstrated the extraction of pharmacologically relevant metabolites, diCQA and CA and their analogues from *B. pilosa*, metabolites known to possess anti-HIV properties [7, 8]. It was observed that although temperature was an important factor to effectively extract these metabolites, extraction efficiency of PHWE can be greatly enhanced by introducing an auxiliary solvent (in this case, methanol). Essentially, it was possible to extract significant amounts of highly unstable metabolites, which is an indicative of an effective extraction method for recovering thermo-labile compounds from plant materials. It was further statistically deduced that solvent composition was a stronger factor that influenced extractability of the target metabolites when compared to temperature. In comparison to the conventional methods of extraction, our modified PHWE method was less time consuming (the total time of extraction being approximately 15 min, whereas solvent extraction takes about 2 h). Moreover, the use of organic solvents was also substantially reduced. The ease and simplicity of the method developed herein is also worthy of note. Once more, the efficacy and applicability of PHWE for the extraction of functional metabolites from plant tissues is reaffirmed, while reiterating the importance of *B. pilosa* and its associated metabolites. Further research could be done using other solvents as alternatives to methanol. Additionally, the synergistic effect of co-solvency and other parameters such as pH on the recovery pattern of metabolites could also be investigated.

## Abbreviations

ANOVA: analysis of variance
BPI: base peak ion
CA: chicoric acid
CCD: central composite design
diCQA: dicaffeoylquinic acid
HIV: human immunodeficiency virus
HPLC: high performance liquid chromatography
ID: inner diameter
*m/z*: mass to charge ratio
MLS: method of least square
MS: mass spectrometry
OD: outer diameter
PHWE: pressurized hot water extraction
qTOF-MS/MS: quadrupole time-of-flight mass spectrometry
RSM: response surface methodology
Rt: retention time
SIM: single ion monitoring
UPLC: ultra-performance liquid chromatography
